# American Public Health Association

**Published:** 1884-08-20

**Authors:** 


### AMERICAN PUBLIC HEALTH ASSOCIATION.

The Twelfth Annual Session of the Association will he held on Tuesday, Wednesday, Thursday and Friday, October 14—17, 1884, at St. Louis, Missouri, and to present the following topics for consideration:

1. Hygiene of the Habitations of the Poor.
2. Hygiene of Occupations.
3. School Hygiene.
4. Adulteration of Food.
5. Water Pollution.
6. Disposal of Sewage by Irrigation or Chemical Action.
7. The Observable Effect upon the Public Health of Official Sanitary Supervision.
8. The work of Municipal and State Boards of Health.

Persons intending to present papers on any of these subjects are requested to notify the Secretary at once, and to furnish him with a condensed abstract of the same not later than September 1st. Members desiring to participate in the discussion of these papers are also requested to inform the Secretary.

